# Pituitary Melanoma Metastasis: A Case Report and Literature Review

**DOI:** 10.7759/cureus.76266

**Published:** 2024-12-23

**Authors:** Énia Sousa, Cícero Silveira, Carolina Camacho, Hugo Dória, Rubina Teixeira, Ricardo Pestana, Pedro Lima

**Affiliations:** 1 Neurological Surgery, Hospital Central do Funchal, Funchal, PRT; 2 Neurosurgery, Hospital Central do Funchal, Funchal, PRT; 3 Oncology, Hospital Central do Funchal, Funchal, PRT; 4 Radiology, Hospital Central do Funchal, Funchal, PRT; 5 Radiation Oncology, Joaquim Chaves Saúde, Funchal, PRT

**Keywords:** malignant melanoma metastasis, melanoma metastasis, pituitary metastasis, sellar masses, sellar metastasis

## Abstract

Metastases to the pituitary gland are a rare finding, with breast and lung being the most common metastases in this anatomical region. Pituitary melanoma metastases reports are thus sparse, and both diagnosis and treatment are challenging. We present the case of a 66-year-old woman with pituitary melanoma metastasis who presented with symptoms of anterior pituitary dysfunction and headache. These symptoms were difficult to manage medically until gross total resection and immunotherapy were carried out. At 17 months post-operative, there was no progression of the underlying disease. A review of the published literature was performed for all the histologically confirmed pituitary melanoma metastases, as well as their clinical presentation, diagnosis, and treatment workout. Our literature review included 18 published cases: the median age was 62, 68% were male, and the median time of melanoma diagnosis to pituitary metastasis diagnosis was 25 months. The most common symptoms were anterior pituitary deficiency, visual disturbances, headache, cranial nerve palsy, and diabetes insipidus. In terms of management, surgery, radiotherapy, and chemotherapy were used alone or in combination. The median overall survival was 9.5 months. These metastases present a great diagnostic and therapeutic challenge since the symptoms are non-specific and there are no available treatment guidelines. Surgery to decompress the adjacent structures allows for symptomatic control and a better quality of life.

## Introduction

Metastases to the pituitary gland occur only in 1% to 3.6% of patients with cancer [1]. Breast and lung neoplasms account for two-thirds of these metastases, followed by prostate and kidney [2]. Pituitary melanoma metastases have been described rarely [1,3-8]. The diagnosis is challenging as, often, patients present with nonspecific signs of systemic malignancy that can overshadow the symptoms of hypopituitarism or diabetes insipidus [2,4]. There is no standardized treatment, and different approaches have been described: surgical resection, radiotherapy, immunotherapy, and chemotherapy [3]. Prognosis is poor [1,9] as they are usually present during the final stages of malignancy [6,7,9,10], with a median survival of 5 to 17 months [2]. The incidence of melanoma is increasing, and cases of metastases to all sites, including the pituitary, will likely become more common [1,4,11]. We present the case of melanoma metastasis to the pituitary and a review of the published literature regarding such metastases, their clinical presentation, and diagnostic and treatment workouts.

## Case presentation

**Figure 1:**
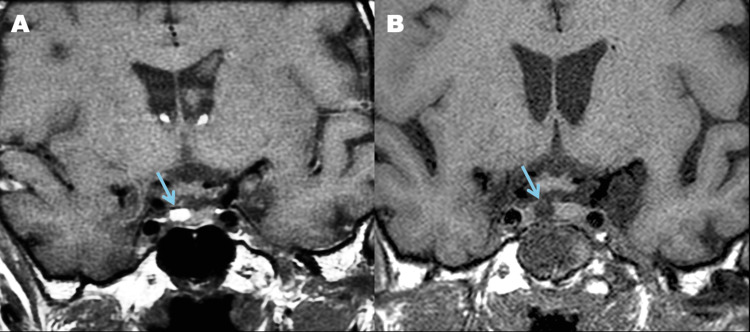
Non-enhanced coronal T1 images before (A) and after (B) procedure. The metastatic lesion exhibited a characteristic high T1 signal (arrow in A). Follow-up MRI showed complete ressection (arrow in B).

We present the case of a 66-year-old woman with a history of skin melanoma that was surgically removed in September 2018 and with lung metastases diagnosed in April 2021 and treated with a tyrosine kinase inhibitor. She presented in August 2022 with headache, nausea, and anorexia. Blood tests showed an abnormal thyroid function that was poorly managed with medical treatment with levothyroxine 88 micrograms daily. Table 1 shows the fluctuation of hormone levels before and after surgery. In November 2022 she was admitted with ionic disturbances and hypocortisolism. An MRI of the pituitary gland showed a round, space-occupying lesion adjacent to the anterior pituitary spontaneously hyperintense in T1 (Figure 1A) and hypointense in T2, in keeping with intracranial metastatic melanoma. In June 2023 the patient underwent an endoscopic transsphenoidal approach to the pituitary, and a gross total resection (GTR) of the dark brown tumor was performed, preserving the adjacent normal gland (Figure 1B). Adjuvant immunotherapy with nivolumab was started. The last follow-up was 17 months after surgery, and she was asymptomatic, with stable endocrinological function under hormonal replacement therapy. There were no signs of disease progression in the brain MRI or body CT scan.

**Table 1:** Blood hormone levels before and after surgery. TSH: thyroid-stimulating hormone

	Pre-operative	Post-operative	Range value
Morning cortisol	0.4 mg/dL	19.9 mg/dL	6.7-22.6 mg/dL
T4	T4 2.8 ng/dL	1.4 ng/dL	0.6-1.7 ng/dL
TSH	0.01 mUI/mL	2.99 mUI/mL	0.3-4.7 mUI/mL

## Discussion

Because pituitary adenomas are common, a sellar tumor in a patient with melanoma might be interpreted as one [4]. With the advances in systemic therapy and prolonged survival of cancer patients, pituitary metastases have been reported with increased frequency in the past decades [11-14] and include melanoma metastases. The imaging appearance of pituitary metastases can mimic that of primary pituitary lesions [13], but metastatic melanoma has a characteristic appearance due to melanin; it is hyperintense on T1-weighted MR images and hypointense on T2-weighted images [5,6,8]. This can be observed in Figure 1A, where the tumor is hyperintense compared to the adjacent pituitary on a T1-weighted image without gadolinium. Clinical signs are no different from other lesions affecting this area [15,16], as these produce disabling symptoms due to the fragile and vital anatomy surrounding the sella. These include headache, visual disturbance, and ophthalmoplegia due to III, IV, and VI cranial nerve palsies, as well as endocrinological disturbances due to the invasion of the gland itself [14-17]. Treatment modalities include radiotherapy, surgical resection, stereotactic radiosurgery, and chemotherapy [17]; however, no management guideline is available. Transsphenoidal surgery had an important role in establishing the diagnosis of pituitary metastasis, but whether it provided a better long-term outcome is uncertain [8]. For cerebral metastatic melanoma, surgery was considered an independent prognostic factor for survival [18].

In our literature review, we included cases of histologically confirmed melanoma metastasis to the pituitary. Only 18 cases of pituitary melanoma metastases have been published; the data can be observed in Table 2; our case would be the 19th. Tumor-to-tumor metastasis, or collision tumor, is present when a tumor metastasis sets on a pre-existing tumor [8,15], for instance, a melanoma metastasis in a pre-existing pituitary adenoma. This was observed in 3 of these 19 cases [5,9,16]. In this literature review, the median age was 62 (range: 32-78), and 13 (68%) were male. The primary site of melanoma was the skin (9), acral (2), stomach (1), and not disclosed (7). The median time of melanoma diagnosis to pituitary metastasis diagnosis was 25 months (range: 0-127). About 14 of these patients didn’t have other intracranial metastases, two had one more intracranial metastasis, and in three it was not disclosed if there were more intracranial mass lesions. The most common symptoms were anterior pituitary deficiency (9), visual disturbances (8), headache (4), cranial nerve palsies (5), diabetes insipidus (4), and pituitary apoplexy (1). In terms of management, eight patients had subtotal resection (SR), seven had GTR, and four had a biopsy. Of these patients submitted to surgery, five had adjuvant immunotherapy, one had chemotherapy, one had combined immunotherapy and chemotherapy, three had radiotherapy, two had chemotherapy and radiotherapy, two had stereotactic radiosurgery and chemotherapy, and five didn’t have any adjuvant treatment. The median survival after pituitary melanoma metastasis diagnosis was 9.5 months (range: 0.3-34.8). These different approaches throughout the published literature are due to an absence of guidelines in the management of this disease.

**Table 2:** Patient characteristics in the included studies by chronological order of publication. ND: not disclosed; SR: subtotal resection; GTR: gross total resection; Bio: biopsy; SRS: stereotactic radiosurgery

	Age (years)	Sex	Primary site of melanoma	Timing to PM (months)	Brain metastases	Symptoms	Surgical treatment	Adjuvant treatment	Last follow-up (months)	Death
Leung et al. (2003) [8]	46	M	Skin	60	No	Diabetes insipidus	GTR	Radiotherapy	7	No
Jung et al. (2007) [16]	75	M	Acral	15	No	Anterior pituitary dysfunction visual field defect	GTR	None	1	Yes
McCutcheon et al. (2007) [4]	77	M	Skin	3	No	Ptosis diplopia	SR	Radiotherapy	6	No
McCutcheon et al. (2007) [4]	42	M	Skin	72	No	Anterior pituitary dysfunction diabetes insupidus	SR	Radiotherapy and chemotherapy	4	Yes
Guzel et al. (2008) [10]	46	F	Skin	84	Yes (1)	Headache	Bio	Radiotherapy and chemotherapy	10	Yes
Kano et al. (2008) [17]	47	M	ND	ND	ND	Diabetes insipidus	Bio	SRS and chemotherapy	34.8	Yes
Kano et al. (2008) [17]	52	F	ND	ND	ND	VI nerve palsy	Bio	SRS and chemotherapy	21.8	Yes
Wang et al. (2011) [1]	78	M	ND	0	Yes (1)	Anterior pituitary dysfunction visual field defect	SR	None	0.3	Yes
Masui et al. (2012) [5]	68	M	Stomach	0	No	Pituitary apoplexy	GTR	None	2	No
Burkhardt et al. (2015) [12]	73	M	ND	0	ND	Anterior pituitary dysfunction visual field defect diabetes insipidus	Bio	Stereotactic radiotherapy (39 Gy)	8	Yes
Yang et al. (2017) [11]	62	F	Skin	24	No	Anterior pituitary dysfunction visual field defect	GTR	None	22	No
Castle-Kirszbaum et al. (2018) [14]	78	M	ND	ND	No	Anterior pituitary dysfunction visual field defect	SR	None	ND	ND
Baiano et al. (2020) [15]	68	F	ND	ND	No	III nerve palsy	SR	Chemotherapy	9	Yes
Mormando et al. (2020) [7]	33	M	Skin	127	No	Headache	SR	Immunotherapy	17	No
Ng et al. (2020) [3]	51	F	ND	0	No	Anterior pituitary dysfunction visual field defect	SR	Immunotherapy	12	Yes
Mattogno et al. (2020) [6]	32	M	Skin	120	No	Ptosis headache	GTR	Immunotherapy	18	No
Mattogno et al. (2020) [6]	32	M	Skin	84	No	III nerve palsy visual field defect	SR	Immunotherapy	14	Yes
Lamorie-Foote et al. (2021) [9]	64	M	Acral	24	No	Anterior pituitary dysfunction	GTR	Chemotherapy and immunotherapy	3	Yes
Present case (2024)	66	F	Skin	57	No	Anterior pituitary dysfunction headache	GTR	Immunotherapy	17	No

## Conclusions

In agreement with the literature, we describe a patient with pituitary melanoma metastasis who presented with symptoms of anterior pituitary dysfunction and headache. These symptoms were difficult to manage with hormone replacement therapy until surgical resection and immunotherapy were performed. At 17 months after surgical intervention, she had no progression of the underlying disease. These metastases present a great diagnostic and therapeutic challenge since there are no available guidelines. Surgery to remove the tumor while preserving the adjacent structures allows for symptomatic control and a better quality of life.
